# “Calibration-on-the-spot”: How to calibrate an EMCCD camera from its images

**DOI:** 10.1038/srep28680

**Published:** 2016-07-06

**Authors:** Kim I. Mortensen, Henrik Flyvbjerg

**Affiliations:** 1 Department of Micro- and Nanotechnology, Technical University of Denmark, Kongens Lyngby, DK-2800, Denmark

## Abstract

In order to count photons with a camera, the camera must be calibrated. Photon counting is necessary, e.g., to determine the precision of localization-based super-resolution microscopy. Here we present a protocol that calibrates an EMCCD camera from information contained in isolated, diffraction-limited spots in any image taken by the camera, thus making dedicated calibration procedures redundant by enabling calibration *post festum*, from images filed without calibration information.

In bio-science and -technology, the nanoscale is investigated with optical microscopy by observing fluorescent probes attached to biological structures of interest. Isolated single molecules are routinely imaged, e.g. in single-particle tracking^1^,^2^,^3^,^4^ and super-resolution microscopy^2^,^5^,^6^. So are distributions of fluorophores, e.g. to track moving biological filaments^7^ or DNA in nanochannels^8^. All these methods overcome the diffraction limit of conventional fluorescence microscopy by fitting theoretical intensity profiles to experimental diffraction-limited intensity distributions that typically are recorded with an electron-multiplying CCD (EMCCD) camera.

An EMCCD camera’s output-signal refers to an arbitrary scale chosen by its user. Calibration of the camera determines its *gain*, the constant that converts detected photon numbers to camera output-signal for a given configuration of the camera. Without calibration it is impossible (i) to compare results across different experimental settings and laboratories, (ii) to determine the precision of localization in an image^2^, and (3) use that precision to discriminate between actual dynamics and fluctuations due to finite statistics on the level of a single measurement^2^,^3^. Here we present a simple, easy, and reliable protocol for calibration of EMCCD cameras. This protocol only uses information contained in a recorded image. Thus it simplifies future data collection by making dedicated calibration procedures redundant. It also makes it possible to determine the gain in old images that were filed without calibration data.

All calibrations of EMCCD-cameras determine the gain, *G*, from the manner pixel output-values scatter about their expected value as function of that expected value. So one can calibrate from any image if one knows which pixels record identical intensities of light. Any set of such pixels have the same expected value, *v*, for the number of photons they register, and hence have the same expected value for their output signals, 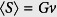. The actual number of photons that falls on any of these pixels is a random integer, Poisson distributed with expected value *v*. The variance with which these random integers scatter about *v* is also *v*; this follows from their Poisson distribution. The stochastic element of the EM amplification makes the variance of the output signals in these pixels Var(*S*) = 2*G*^2^*v*, which is a factor two larger than what would result from deterministic amplification—this factor two is due to the *excess noise*^2^,^9^,^10^ of the electron-multiplication process, which adds to the Poisson noise of the photon number, a.k.a. *photonic shot noise*. The gain now seems known through 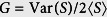. In practice, however, an EMCCD camera^9^,^10^,^11^ is designed to ensure positive output values in the presence of Gaussian read-out noise by having a constant *offset*, *S*_offset_, added to all its output signal values (Supplementary Note). So


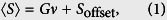


and


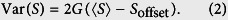


Hence, the variance Var(*S*) of the output signal from pixels exposed to the same intensity of light grows as a first-degree polynomial in their expected output-signal 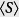. Thus, a plot of *experimental estimates* for such variances against corresponding *experimental mean values* will scatter about a straight line with slope 2*G* that intersects the first axis at *S*_offset_. Given such data for a range of light intensities, the parameters *G* and *S*_offset_ in equation 2 are determined by fitting a straight line to these data^12^,^13^.

## Results

### Methodology of calibration-on-the-spot

**Figure 1:**
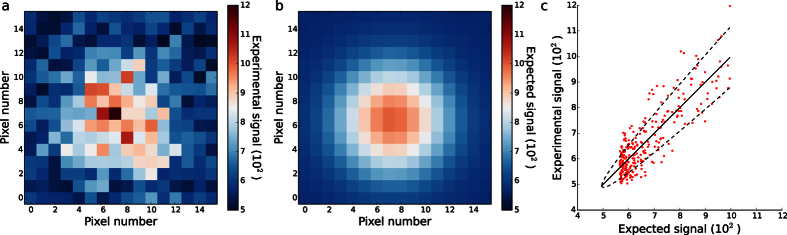
Protocol for calibration-on-the-spot. (**a**) TIRF microscopy with effective pixel size 44 nm imaging an individual rhodamine fluorophore (Methods). (**b**) Theoretical image with parameter values obtained by fitting the theoretical PSF to the image in **a** using the Gaussian Mask Estimator^2^,^4^ (Methods). This determined the expected values of pixel output-signals. (**c**) For each pair of corresponding pixels in (**a**,**b**), the experimental signal from **a** is plotted against the expected signal from (**b**) (red points). The points scatter around the straight line through the origin having unit slope (full line), and the residuals at each expected value scatter with variance given in equation (2), with an approximately normal distribution. With the expected output signals already determined from the localization analysis in (**b**), this scatter allows calibration of the EMCCD camera parameters, as described in the main text. For this particular image, we found a gain *G* = 14 ± 3 and signal offset *S*_offset_ = 500 ± 100. These values have been corrected for bias (Methods). Using these parameters, dashed lines indicate ± s.d. as calculated from equation (2).

In the logic outlined above, it is actually not necessary to have many pixels exposed to the same intensity of light for each intensity used in the calibration. One pixel is enough. What matters is that one knows the expected output from a large number of pixels. Knowing that, the mean squared deviation between actual and expected output from a single pixel is an estimate of the variance of the output from that pixel. It is a rough estimate, but by combining many of these, a fine calibration results. So if one can estimate the expected output-signals for many pixels in an image, covering a range of intensities, one can calibrate an EMCCD camera from such an image. This one can do in images containing isolated point sources of light (Fig. 1a), be they fluorescent probes or distant stars. What matters is that isolated point-sources image as isolated diffraction limited spots, and that one knows the point spread function (PSF) for such sources. We fit the appropriate PSF to such a diffraction-limited spot, after which it tells us the expected output-signal in each pixel in the diffraction-limited spot it was fitted to. Applying the logic above, we calibrated the camera by estimating Var(*S*) for each pixel in a spot by 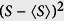, where *S* is the actual output-signal and 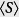 is our estimate for the expected output-signal. We refer to this protocol as “calibration-on-the-spot” for this reason, and because the calibration achieved is the one that was valid for the camera in the instant that the image was taken. In practice, for better statistics, one fits PSFs to several different isolated point sources in an image and/or to different images of the same spot imaged as a time-lapse movie.

For demonstration, localization microscopy provides a pertinent example: An isolated fluorescent probe images as a diffraction-limited spot (Fig. 1a) with an intensity distribution that often may be approximated well by a 2-dimensional (2D) Gaussian plus a constant “background”^2^ (Methods, Supplementary Fig. 1). This theoretical point spread function (PSF) is routinely fitted to such experimental spots to localize probes^1^,^2^,^3^,^4^,^5^,^6^.

The optimal statistical procedure to this end is maximum likelihood estimation (MLE), but MLE requires known photon statistics, i.e., it requires a calibrated camera^2^. Ordinary least-squares fitting of the PSF to the image works fine with unknown photon statistics/camera calibration. It results in the so-called Gaussian Mask Estimator^2^,^4^ (GME, Methods, Supplementary Note), which is sub-optimal^2^. But once calibration has been performed with GME as described below, the PSF can be fitted again, using MLE for optimal precision^2^. Thus, suboptimal fitting is the gateway to optimal fitting.

Using GME for localization of the fluorophore imaged in Fig. 1a also yields the width of the spot, the total source intensity, and the constant background (Methods). The PSF with these parameters defines a theoretical image (Fig. 1b). In this theoretical image, the value in each pixel is our estimate for the pixel’s expected output-signal. This estimate is relatively well determined statistically, because it depends only on the few parameters of the theoretical PSF, and they were determined by a fit to all experimental pixel output-signals in the spot shown. The experimental values (Fig. 1a) scatter around their expected values (Fig. 1b) with s.d. given by the square root of equation (2) (Fig. 1c). This and the variation in expected signal across a diffraction-limited spot (Fig. 1b,c), is sufficient to determine both parameters of the EMCCD camera from just a single image of the spot (Fig. 1c, Methods).

Since we do not know 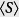, but estimate it, our estimates for Var(*S*) as function of 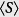 are *conditional averages*, which slightly complicates the determination of the calibration parameters from pairs of these estimates. It affects our estimates for *G* and *S*_offset_ with biases that depend in magnitude on the number of pixels covering a spot but has negligible effects on their precision (Supplementary Note and Supplementary Figs 2–4). With small pixels (<40 nm), these biases are insignificant (<10 percent) for most purposes, but for situations where they are not and for use of calibration-on-the-spot with experimentally relevant larger pixels (<70 nm), we give their values analytically (Supplementary Note) and used them to correct for bias (Fig. 1c and Supplementary Figs 2–5). This makes calibration-on-the-spot accurate and precise over a large parameter space (Methods, Supplementary Figs 3–5).

### Calibration performance using single-molecule data

**Figure 2:**
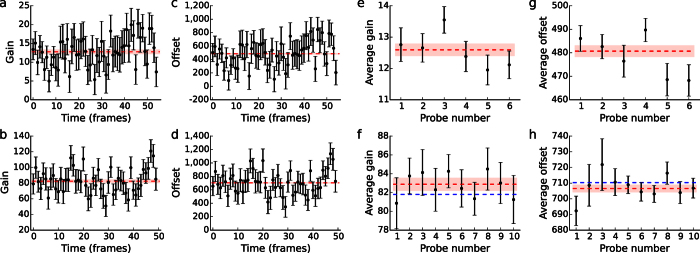
Performance of calibration-on-the-spot. (**a**) The EMCCD camera’s gain (black points) was estimated using calibration-on-the-spot for each frame of a time-lapse movie of single rhodamine fluorophores labeling myosin V molecules that were stepping along actin filaments. Error bars represent s.d. as calculated from the theoretical covariance matrix (Methods, Supplementary Note). The time-averaged gain was *G* = 12.8 ± 0.5 (mean ± theoretical s.e.m., red dashed line with shaded area). (**b**) Same as **a** for a time-lapse movie of a single Cy3 fluorophore. The time-averaged gain of that camera was *G* = 82 ± 2. (**c**) The EMCCD camera’s offset (black points) was estimated using calibration-on-the-spot simultaneously with the estimates in (**a**). The time-averaged offset was *S*_offset_ = 486 ± 5 (mean ± theoretical s.e.m., red dashed line with shaded area). (**d**) Same as **c** but corresponding to the data in **b**. The time-averaged offset was *S*_offset_ = 704 ± 6. (**e**) Time-averaged gains (black points) obtained as in **a** for six myosin molecules. Error bars represent s.e.m. as calculated from the theoretical covariance matrix (Methods). The weighted average over the molecules was *G* = 12.6 ± 0.2 (mean ± theoretical s.e.m., red dashed line with shaded area). (**f**) Same as **e** for ten Cy3 fluorophores obtained as in (**b**). In this case, the weighted average was *G* = 82.9 ± 0.7. This value agrees well with the value (blue dashed line) obtained using an alternative calibration method (Methods, Supplementary Fig. 8 and Supplementary Note). (**g**) Same as **e** for time-averaged offsets obtained as in **c**. The weighted average offset calculated over the molecules was *S*_offset_ = 481 ± 2. (**h**) Same as **f** for time-averaged offsets obtained as in (**d**). Here, the weighted average was *S*_offset_ = 707 ± 2. In all cases (**a–h**), the estimates scatter around their respective mean values as dictated by the theoretical error bars, demonstrating that all variation is accounted for by finite photon statistics and the EMCCD’s excess noise (Supplementary Fig. 7) and therefore that the estimates of the camera’s calibration parameters are optimally precise.

To illustrate the performance of the method, we calibrated an EMCCD camera repeatedly, from each frame in time-lapse movies of (i) single rhodamine fluorophores, each attached to a molecular motor, myosin V, that moved processively along actin^1^, and (ii) single Cy3 fluorophores immobilized on the coverslip surface but free to rotate (Methods). These movies were recorded with total internal reflection fluorescence (TIRF) microscopy. In each frame of these movies, we localized the isolated fluorophores using GME and then applied calibration-on-the-spot, as described above, to determine the camera parameters as *G* and 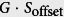, respectively (Methods). This combination of parameters appears linearly in equation (2), which ensures good convergence properties for their estimates, as single-frame estimates for the gain scattered around common constant values with normal distributions (Fig. 2a,b), and so did the estimates for the product of the gain and the offset (Supplementary Fig. 6). In each case, the scatter had an s.d. given by the theoretical covariance matrix for the estimates (Fig. 2a,b and Supplementary Figs 6 and 7). We then used the average gain to calculate single-frame estimates of the signal offset. They also scattered around a common constant value (Fig. 2c,d) with an s.d. calculated from the theoretical covariance matrix for those estimates (Fig. 2c,d and Supplementary Fig. 7). This analysis demonstrates that the fluctuations in the estimates are fully accounted for by our finite statistics, and hence that calibration-on-the-spot is optimally precise (Methods).

We repeated this analysis sixteen times: for six myosin motor molecules and for ten single Cy3 fluorophores. The time-averaged calibration parameters for each experiment scatter around constant values (Fig. 2e–h), which is expected when all probes in each experiment have been recorded with the same camera settings. Furthermore, for each experiment, the scatter of each time-averaged quantity is fully explained by our finite statistics. This demonstrates that calibration-on-the-spot provides single-molecule results and consistently so from molecule to molecule.

We compared the calibration parameters obtained for the Cy3 probes using calibration-on-the-spot to values obtained using an alternative calibration procedure^2^,^3^,^11^ (Methods, Supplementary Fig. 8 and Supplementary Note) and found agreement within one per cent, fully consistent with the precision of our results from calibration-on-the-spot (Fig. 2f,h, Methods). This agreement between the two calibrations indicates that calibration-on-the-spot provides accurate estimates for calibration parameters in an experimental setting. This alternative calibration procedure was not available for the myosin data set (Fig. 2e,g), as suitable regions were not imaged in those movies. This left calibration-on-the-spot as the only way to calibrate *post festum*.

As an additional demonstration, and to show that the analysis also works for other fluorescent probes, we repeated the analysis for a single 40-nm fluorescent bead imaged with a TIRF microscope (Methods). In this case we determined time-averaged calibration parameters from 500 frames in a time-lapse movie with less than one per cent error (Supplementary Fig. 9).

## Discussion

Although the data presented here demonstrate the performance of the method in the context of localization-based microscopy and EMCCD cameras, the method must work for other applications that use other theoretical intensity distributions^2^,^3^,^7^,^8^ and/or detectors^13^, and its application should be straight-forward.

Calibration-on-the-spot in its simplest form is implemented with just a few extra lines of code (Methods) in the localization software used in a given laboratory. Supplementary Software presents an implementation of calibration-on-the-spot that corrects for bias due to conditional averaging and calculates the variance-covariance matrix for the estimates.

With calibration-on-the-spot, data sets already on file may be calibrated or re-calibrated now. Irrespective of whether the cameras still exist, calibration-on-the-spot calibrates it for the state it was in at the instant it recorded the data. Moreover, calibration-on-the-spot is so accurate and precise that each snapshot of an isolated fluorescent probe may be analyzed independently. This allows for elimination of possible outlying calibration measurements on the single-snapshot level before data are pooled into averages^14^. Calibration-on-the-spot also provides an easy-to-use method for future data acquisition, since a separate calibration experiment can be skipped: all information necessary for calibration is already encoded in experimental images and may be extracted as demonstrated here. Separate calibration experiments can also be skipped with the latest generation of EMCCD cameras, which can calibrate themselves. But the experimental and/or data-analytic protocols of this functionality may differ between vendors, which may complicate comparison of experiments. In contrast, calibration-on-the-spot calibrates independently—independent of cameras, camera settings, experiments, and laboratories—it constitutes a *lingua franca* of calibration.

## Methods

### Localization analysis

We selected isolated, diffraction-limited spots in microscope images (Fig. 1a). Typically, we ensured that a single, fluorescent probe produced each spot, by verifying that the spot intensity remained constant in time until it photo-bleached in a single step. For analysis, we used pixels in a square region around each spot (Fig. 1a), where the region’s size was chosen such that most of the “shoulders” of the theoretical PSF were included (Supplementary Fig. 1). This choice ensured that a 2D-Gaussian-plus-a-constant-background PSF accurately approximates the theoretical PSF (Supplementary Fig. 1) and in turn that calibration-on-the-spot calibrates with accuracy and precision (Supplementary Figs 2–5).

Without a calibration of the EMCCD camera, the output statistics of individual pixel signals are unknown, precluding use of maximum likelihood estimation (MLE). Instead, we used the so-called Gaussian Mask Estimator (GME)^2^,^4^, which results from applying unweighted least-squares estimation in conjunction with a 2D-Gaussian-plus-a-constant-background as a model for the PSF (Supplementary Note). This estimator is sub-optimal^2^, however, because it ignores the weights of the contributions of the individual pixels in the localization analysis. In a practical setting, the localization analysis should therefore be repeated using MLE, which is optimal^2^, once the calibration of the EMCCD camera has been performed.

In the application of GME to each frame in a time-lapse movie of an isolated spot (Fig. 1b), we estimated five parameters: the two coordinates of the location of the probe, the width of the PSF, the expected total detected signal, the expected constant “background” signal. In this process, we also recorded (i) the expected value of the pixel output signals, obtained from the theoretical image (Fig. 1b); and (ii) the values of a function that describes how statistical fluctuations of experimental pixel output-signals affect the fluctuations in the fitted, theoretical pixel output-signal of any given pixel (Supplementary Note). The latter is used only to calculate the covariance matrix of the calibration parameters estimated using calibration-on-the-spot and to correct for bias.

### Protocol for calibration-on-the-spot

For each pixel, we assumed that the experimental output-signal value is normally distributed around its true output-signal value with a variance given by equation (2) (Supplementary Fig. 10 and Supplementary Note). The validity of this assumption increases with larger incident photon number but was typically satisfactory everywhere in images because of background fluorescence and the fact that the “shoulder” of the theoretical PSF is interpreted as additional background in the localization analysis (Methods, Supplementary Fig. 1).

For each pixel, the true output-signal is inherently unknown, so in its place we used the fitted expected value obtained from the localization analysis. Note that the expected pixel output-signal itself is an explicit function of the calibration parameters (Supplementary Note). However, none of its parameters may be independently determined without a calibration, only its value may (Supplementary Note). Therefore, in the methodology of calibration-on-the-spot, this dependence on the calibration parameters is immaterial and for the same reason, the localization analysis may be done prior to the calibration rather than jointly with it. Thus, only the explicit dependence on the gain and the signal offset in equation (2) matters for calibration. The latter we parameterized as 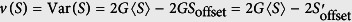, with 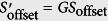, and proceeded to initially estimate, respectively, *G* and 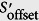 in individual images of a spot. Estimation with this parameterization ensured superior convergence properties, because the fitted function depends linearly on these two parameters.

Thus, to calibrate using MLE, we maximized the log-likelihood


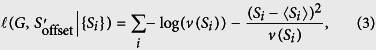


with respect to the parameters *G* and 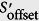 (Supplementary Note). Here the summation is over all pixels in the region of the image around the spot (Fig. 1a and Supplementary Fig. 2a) and it is understood that fitted expected pixel output-signals, known from the localization analysis, replace their true values 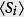 everywhere. This determined the parameters *G* and 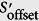 from single images (Fig. 2a,b and Supplementary Figs 2,6 and 9).

The use of the fitted expected pixel output-signal values instead of their true values, above, introduces a bias in the estimated parameters. The magnitude of this bias increases with the pixel size, because fewer pixels then cover a spot. For small pixels (<40 nm), however, it remains below 10 percent (Supplementary Fig. 5). For applications where this bias cannot be ignored, and for larger pixels (<70 nm), we proceeded to calculate the bias analytically and used that result to correct for bias (Supplementary Fig. 5). To this end, we initially determined the calibration parameters *G* and 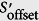 from either a single image or all images in a time-lapse movie. We then used their (time-averaged) values and the fitted expected pixel output-signal values from the localization analysis (Methods) to calculate corrections for each frame (Supplementary Note). Then, we corrected the values of each estimate for *G* and 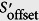 before we used them to calculate *S*_offset_.

### Covariance matrix for parameter estimates

Similarly, we calculated the theoretical covariance matrix for each calibration-on-the-spot estimate of the parameters *G* and 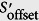 (Supplementary Note). Using this, the theoretical uncertainties for the time-averaged values of *G* and *S*_offset_ were found by propagation of errors. All single-frame variation in parameter estimates is explained by the theoretical uncertainty as calculated from the theoretical covariance matrix (Fig. 2a–d and Supplementary Figs 2,3,6 and 9), demonstrating that calibration-on-the-spot is optimally precise.

For single image calibrations (Fig. 1), the offset 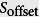 is found directly from the estimated parameters by division as 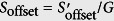. For time-lapse movies (Fig. 2), on the other hand, initially, we found the single-frame values of *G* (Fig. 2a,b and Supplementary Figs 2 and 9) and 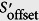 (Supplementary Figs 2,6 and 9) and then used the time-average of 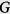 to estimate the single-frame estimates for 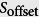 from the single-frame estimates of 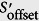 (Fig. 2c,d), but we used the time-averaged values of both *G* and 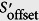 to calculate reported (time-averaged) values for 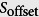. Because the theoretical errors themselves are calculated based on the estimated calibration parameters, we avoided bias in the calculation of the time-averaged calibration parameters, by calculating these as unweighted averages over individual frames in Fig. 2a–d and Supplementary Figs 2,6 and 9. On the other hand, the errors on these time-averaged estimates were so well determined that we calculated the weighted averages of the calibration parameters from different probes in Fig. 2e–h and Supplementary Figs 2 and 4.

### Simulations

To simulate images, we used the theoretical PSF for a freely rotating fluorescent probe, as previously described^2^. For each pixel in an image, we calculated the probability of detecting a photon there as the integral of the PSF over the area of that pixel. Multiplication with the expected total photon number and addition by the number of expected background photons per pixel yielded the expected number of photons recorded by that pixel. With this expected value, we generated a Poisson distributed random number, to simulate the number of detected photons in that pixel. Based on this, an Erlang distributed random number modeled the amplification process in the EMCCD camera, and with addition of the constant signal offset and a normally distributed random number to model readout-noise distribution, we simulated a pixel output-signal value (Supplementary Figs 2 and 3 and Supplementary Note).

To demonstrate that the assumption of normally distributed pixel output-signal values did not significantly compromise calibrations with calibration-on-the-spot, we also generated images using normally distributed pixel output-signals with moments given by Eqs (1) and (2). This modelled the amplification process. We assessed the performance of calibration-on-the-spot in this approximation (Supplementary Figs 3–5). The assumption of a 2D-Gaussian-plus-a-constant-background PSF assumed in GME did not significantly compromise calibrations, we demonstrated with simulations using a 2D-Gaussian-plus-a-constant-background as the PSF (Supplementary Figs 3–5). For those simulations, we adjusted the 2D-Gaussian’s total photon number as well as the background, so they agree with the PSF for a freely rotating probe (Supplementary Fig. 1).

### Source of experimental data

We assessed the applicability and performance of calibration-on-the-spot on sets of experimental data graciously shared by Professor James A. Spudich’s laboratory (Stanford University School of Medicine). The laboratory provided TIRF microscopy time-lapse movies recorded with an EMCCD camera (Andor Technology; iXon DV 887 EMCCD) of (i) the processive molecular motor myosin V labeled with a rhodamine fluorophore and stepping along actin filaments on the coverslip surface. The effective EMCCD pixel size was 44 nm and the average emission wavelength was 580 nm. The time-lapse movies were recorded at 5 Hz; (ii) single Cy3 fluorophores immobilized on the coverslip surface. The effective camera pixel size was 28 nm and the average emission wavelength was 580 nm; and (iii) a 40-nm fluorescent bead immobilized on the coverslip surface, as previously described^2^. The effective pixel size was 28 nm and the peak emission wavelength was 605 nm.

## Additional Information

**How to cite this article**: Mortensen, K. I. and Flyvbjerg, H. “Calibration-on-the-spot”: How to calibrate an EMCCD camera from its images. *Sci. Rep.* **6**, 28680; doi: 10.1038/srep28680 (2016).

## Supplementary Material

Supplementary Information

SupplementarySoftware_readme

SupplementarySoftware

SupplementarySoftware_test

SupplementarySoftware_testdata

## References

[b1] Yildiz A. . Myosin V walks hand-over-hand: single fluorophore imaging with 1.5-nm localization. Science 300, 2061–2065 (2003). 12791999 10.1126/science.1084398

[b2] Mortensen K. I., Churchman L. S., Spudich J. A. & Flyvbjerg H. Optimized localization analysis for single-molecule tracking and super-resolution microscopy. Nat. Methods 7, 377–381 (2010). 20364147 10.1038/nmeth.1447 PMC3127582

[b3] Mortensen K. I., Sung J., Flyvbjerg H. & Spudich J. A. Optimized measurements of separations and angles between intra-molecular fluorescent markers. Nat. Commun. 6, 8621 10.1038/ncomms9621 (2015). 26509412 PMC4634324

[b4] Thompson R. E., Larson D. R. & Webb W. W. Precise nanometer localization analysis for individual fluorescent probes. Biophys. J. 82, 2775–2783 (2002). 11964263 10.1016/S0006-3495(02)75618-X PMC1302065

[b5] Betzig E. . Imaging intracellular fluorescent proteins at nanometer resolution. Science 313, 1642–1645 (2006). 16902090 10.1126/science.1127344

[b6] Rust M. J., Bates M. & Zhuang X. W. Sub-diffraction-limit imaging by stochastic optical reconstruction microscopy (STORM). Nat. Methods 3, 793–795 (2006). 16896339 10.1038/nmeth929 PMC2700296

[b7] Ruhnow F., Zwicker D. & Diez S. Tracking single particles and elongated filaments with nanometer precision. Biophys. J. 100, 2820–2828 (2011). 21641328 10.1016/j.bpj.2011.04.023 PMC3117161

[b8] Tegenfeldt J. O. . The dynamics of genomic-length DNA molecules in 100-nm channels. Proc. Natl. Acad. Sci. 101, 10979–10983 (2004). 15252203 10.1073/pnas.0403849101 PMC503729

[b9] Hynecek J. & Nishiwaki T. Excess noise and other important characteristics of low light level imaging using charge multiplying CCDs. IEEE Trans. Electron Devices 50, 239–245 (2003).

[b10] Robbins M. S. & Hadwen B. J. The noise performance of electron multiplying charge-coupled devices. IEEE Trans. Electron Devices 50, 1227–1232 (2003).

[b11] Ulbrich M. H. & Isacoff E. Y. Subunit counting in membrane-bound proteins. Nat. Methods 4, 319–321 (2007). 17369835 10.1038/NMETH1024 PMC2744285

[b12] Lidke K. A., Rieger B., Lidke D. S. & Jovin T. M. The role of photon statistics in fluorescence anisotropy imaging. IEEE Trans. Image Process. 14, 1237–1245 (2005). 16190460 10.1109/tip.2005.852458

[b13] Huang F. . Video-rate nanoscopy using sCMOS camera-specific single-molecule localization algorithms. Nat. Methods 10, 653–658 (2013). 23708387 10.1038/nmeth.2488 PMC3696415

[b14] Vestergaard C. L., Pedersen J. N., Mortensen K. I. & Flyvbjerg H. Estimation of motility parameters from trajectory data. Eur. Phys. J. Spec. Top. 224, 1151–1168 (2015).

