# Erratum: “Calibration-on-the-spot”: How to calibrate an EMCCD camera from its images

**DOI:** 10.1038/srep46883

**Published:** 2017-09-06

**Authors:** Kim I. Mortensen, Henrik Flyvbjerg

Scientific Reports 6: Article number: 28680; 10.1038/srep28680 published online: 07 06 2017; updated: 09 06 2017.

In the original version of this Article, the Supplementary Software files were omitted. This has been corrected in the HTML version of the Article; the PDF version was correct at the time of publication.

