# Supplementary material for: “Calibration-on-the-spot”: How to calibrate an EMCCD camera from its images
Source: Sci Rep. 2016 Jul 6;6:28680. doi: 10.1038/srep28680 (PMC4933971; doi:10.1038/srep28680)
Supplement: Supplementary Information [file srep28680-s1.pdf]

SUPPLEMENTARY INFORMATION

for

"Calibration-on-the-spot": How to calibrate an EMCCD camera from its images

Kim I. Mortensen[+]* and Henrik Flyvbjerg[+]

[+] Department of Micro- and Nanotechnology, Technical University of Denmark, Kongens Lyngby, DK-2800, Denmark

* Corresponding author

SUPPLEMENTARY FIGURES

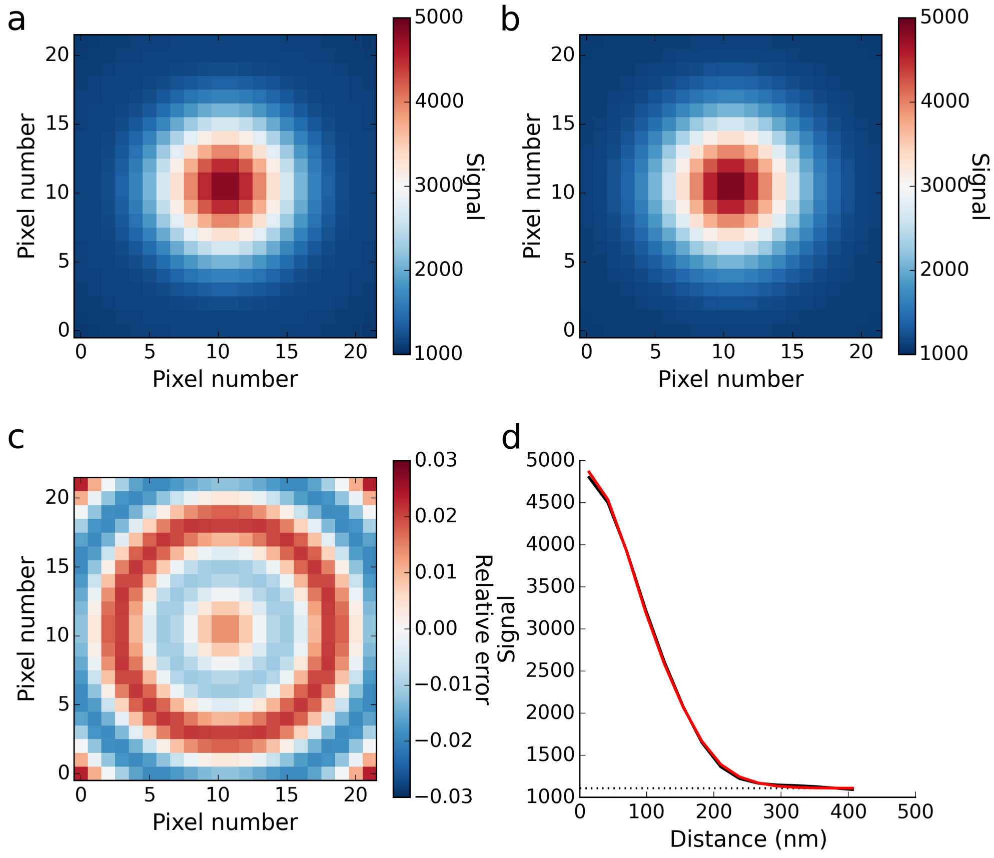

Supplementary Figure 1

The PSF for a single, freely rotating fluorophore. (**a**) The theoretical PSF of a freely rotating fluorophore imaged with TIRF microscopy in focus and close to the coverslip, as reported previously[1]. For its calculation, we used the experimental parameters relevant for the experiments with Cy3 reported in the main paper (Methods). The effective pixel size was 28 nm. Here we used a total photon number of 10,000 and assumed absence of photonic background. For the EMCCD parameters (Supplementary Note), we used a gain of 80 and a constant signal offset of 1,000. (**b**) For comparison, a 2D Gaussian plus-a-constant-background PSF with parameters obtained by using GME (Methods, Supplementary Note) on the image in **a**. (**c**) Relative error between the PSFs in **a** and **b**. Everywhere in the image, the 2D-Gaussian-plus-a-constant-background approximates the theoretical PSF of a freely rotating fluorophore with discrepancies of only a few per cent. Note that the discrepancies are symmetric about the centre of the PSF. Note also that the largest relative discrepancies occur where the absolute value of the PSF is small and slowly changing in space. Consequently, the discrepancies contribute little to localization errors. (**d**) Comparison of radial distributions of the theoretical PSF (black) and the 2D-Gaussian-plus-a-constant-background (red). To calculate these, we constructed a

set of concentric and equally spaced annuli around the centre. Each pixel in the images in **a** and **b** was associated with an annulus, if the pixel's centre fell within the annulus. For each annulus, we calculated the average signal over signals from pixels associated with it and plotted this as a function of the distance from the image centre to the centre of the annulus. This reveals a "shoulder" in the theoretical distribution as indicated by the dotted line. The 2D-Gaussian-plus-a-constant-background PSF approximates the full theoretical model well by modelling the "shoulder" as "background".

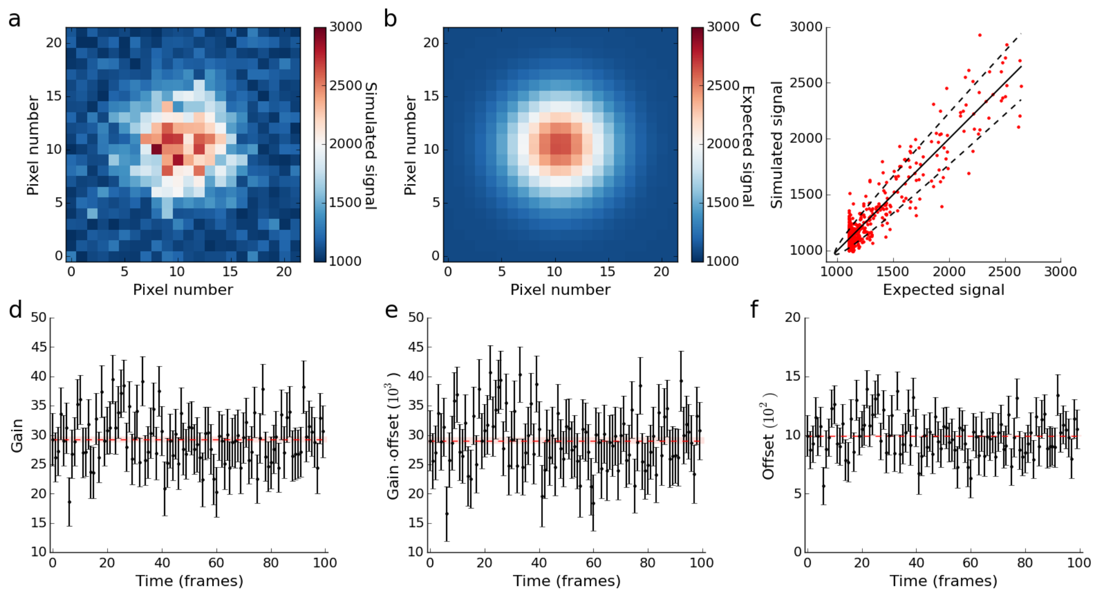

Supplementary Figure 2

Calibration-on-the-spot applied to simulated images. (**a**) Simulated image of a single, freely rotating fluorophore. The pixel output-signal values were simulated using EMCCD camera statistics for the pixel output-signals (Methods). The pixel size was 30 nm, the gain was 30, and the constant offset was 1,000. (**b**) Theoretical image of a 2D-Gaussian-plus-a-constant PSF with parameters obtained from applying GME (Methods, Supplementary Note) to the simulated image in **a**. (**c**) For each pixel in the corresponding images **a** and **b**, the simulated output-signal from **a** is plotted versus the fitted expected pixel output-signal from **b**. The points scatter around a straight line through the origin with unit slope (full line), as expected if the assumed PSF is a good approximation of the true PSF. The scatter of the points relative to the line has a variance given by Eq. (2) in the main paper. Maximum likelihood estimation applied to the scatter yields the calibration-on-the-spot estimate for the calibration parameters (Methods). The s.d. of the scatter with the estimated calibration parameters is indicated (dashed lines). (**d**) For each of 100 images as in **a**, the gain was estimated using calibration-on-the-spot (Methods, Supplementary Note). The values were corrected for bias (Methods, Supplementary Note). Error bars represent s.d. as calculated from the theoretical covariance matrix (Methods, Supplementary Note). The time-averaged gain is indicated (mean ± theoretical s.e.m., red dashed line with shaded region). (**e**) Same as **d** for the product of the gain and the offset. (**f**) Same as **d** for the offset found from division of the frame-to-frame estimates of the product of gain and offset in **e** by the time-averaged value of the gain obtained in **d**.

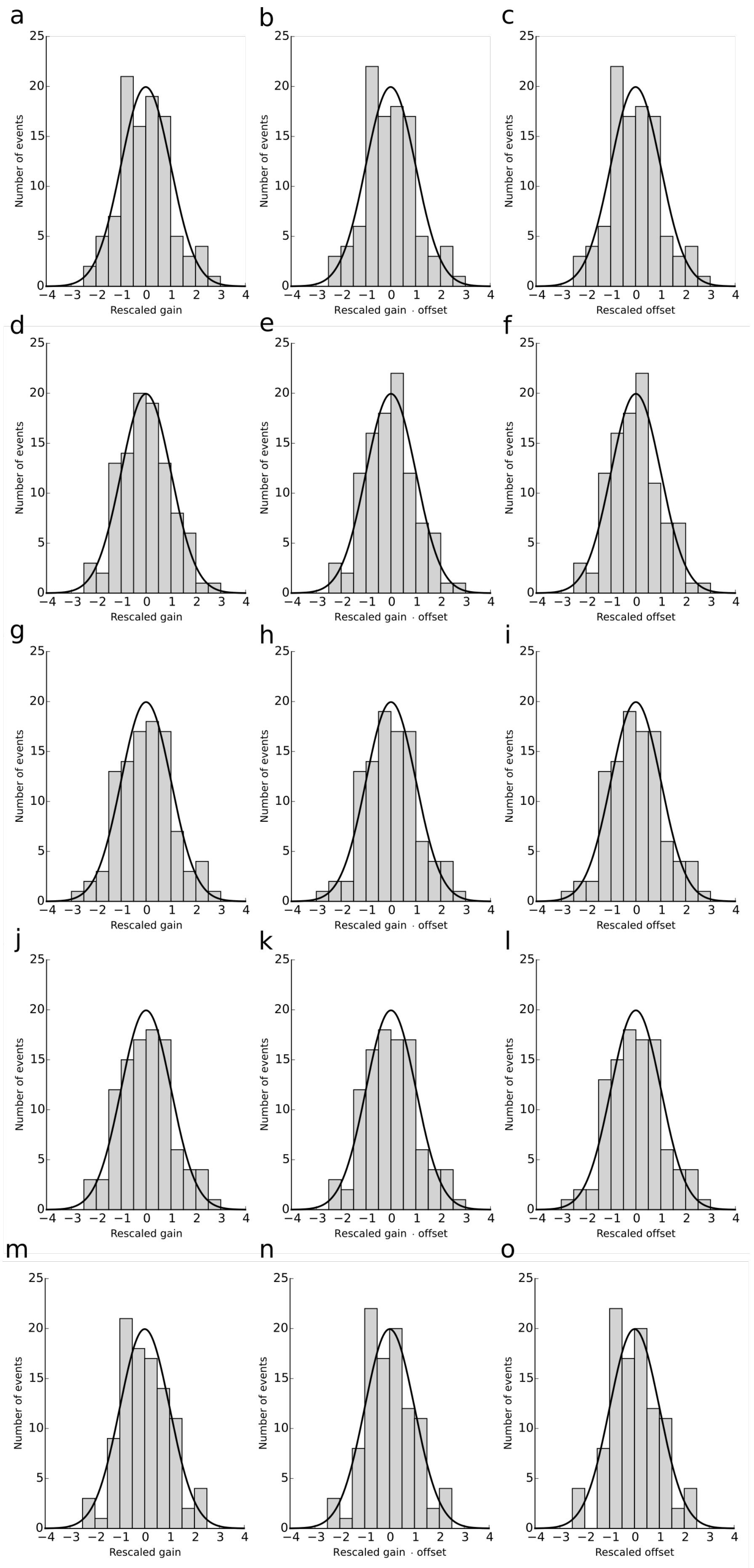
a
Number of events
Rescaled gain
b
Number of events
Rescaled gain · offset
c
Number of events
Rescaled offset
d
Number of events
Rescaled gain
e
Number of events
Rescaled gain · offset
f
Number of events
Rescaled offset
g
Number of events
Rescaled gain
h
Number of events
Rescaled gain · offset
i
Number of events
Rescaled offset
j
Number of events
Rescaled gain
k
Number of events
Rescaled gain · offset
l
Number of events
Rescaled offset
m
Number of events
Rescaled gain
n
Number of events
Rescaled gain · offset
o
Number of events
Rescaled offset

Supplementary Figure 3

Precision of calibration-on-the-spot. We repeated the simulations described above (Supplementary Fig. 2) for movies consisting of 100 frames. We also simulated data corresponding to various approximations in the methodology of calibration-on-the-spot. (**a**) For the data in Supplementary Fig. 2d, we subtracted the time-averaged gain from the frame-to-frame estimates of that quantity and rescaled each of those with their theoretical r.m.s. deviation. Their distribution is normal with zero mean and unit variance (black curve), demonstrating that all variation in the estimates is accounted for by finite statistics, and that calibration-on-the-spot is optimally precise. (**b**) Same as **a** for the product of gain and offset from Supplementary Fig. 2e. (**c**) Same as **a** for the offset from Supplementary Fig. 2f. (**d-f**) Same as **a-c** for data simulated assuming a 2D-Gaussian PSF with parameters matching those of the PSF for the freely rotating fluorophore (Methods, Supplementary Figs. 1-2) and pixel output-signals distributed according to EMCCD camera statistics. (**g-i**) Same as **d-f** but assuming normally distributed output-signals (Methods, Supplementary Fig. 10). (**j-l**) Same as **g-i** but here the known true values of the expected pixel output-signals and calibration parameters were used in the correction for bias. There is no discernible difference between **j-l** and **g-i**, demonstrating that we introduce negligible error by using the fitted values for the expected pixel output-signals and the calibration parameters. (**m-o**) Same as **j-l** but using the known expected values of pixel output-signals in the fit. This artificially eliminates the need for the localization analysis and thus eliminates the bias introduced by that (Supplementary Note), hence no bias correction was applied here.

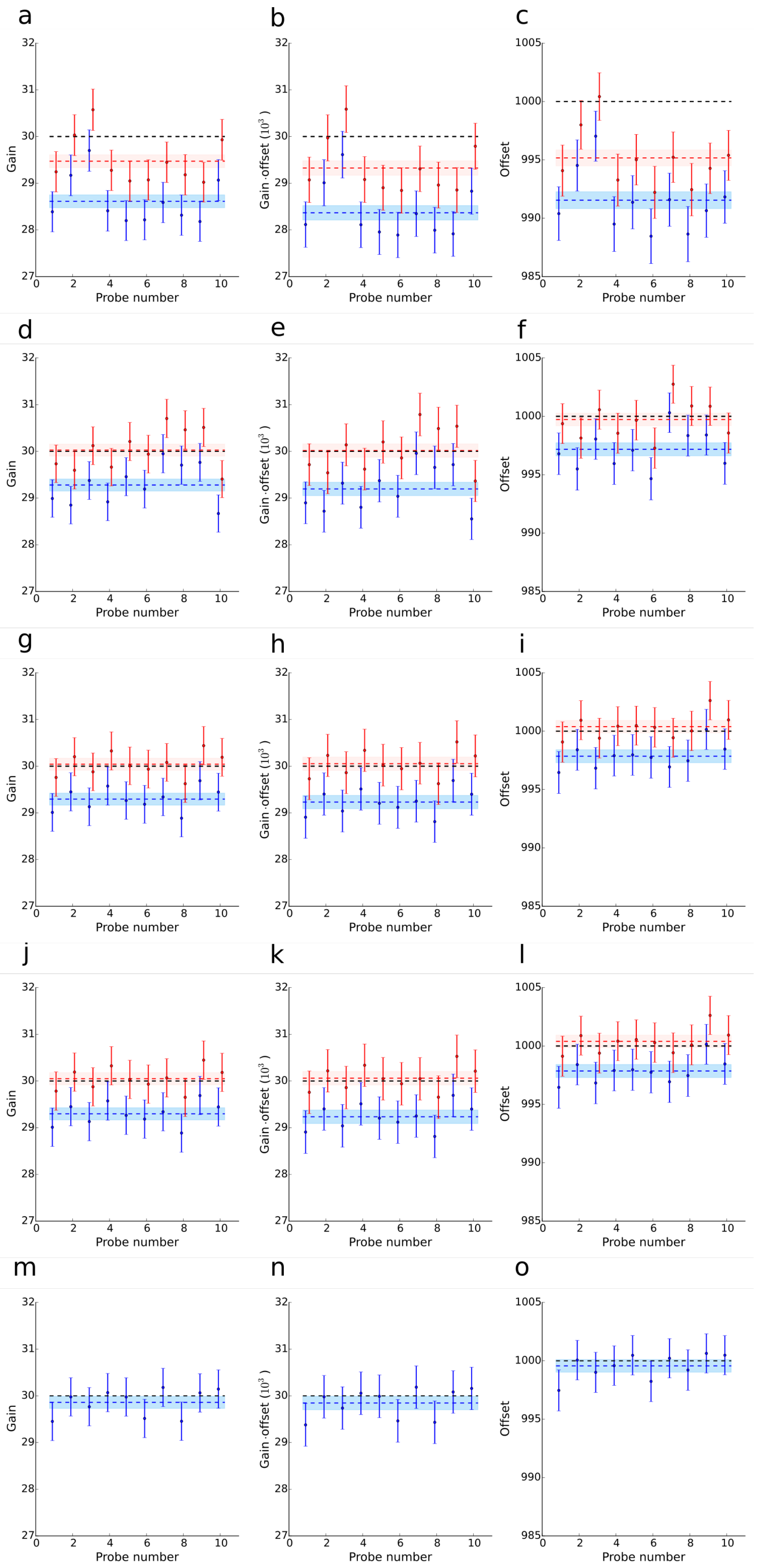

a
b
c
d
e
f
g
h
i
j
k
l
m
n
o
Gain
Gain·offset (10³)
Offset
Probe number

Supplementary Figure 4

Accuracy of calibration-on-the-spot. We repeated the simulations described above (Supplementary Figs. 2 and 3) for movies consisting of 100 frames for ten probes. (**a**) Time-averaged gains (red circles) obtained using calibration-on-the-spot on such data simulated by assuming the PSF for a freely rotating fluorophore (Supplementary Fig. 1) and pixel output-signals distributed according to EMCCD camera statistics (Methods, Supplementary Note). Error bars are theoretical s.e.m. and the estimates were corrected for bias (Methods, Supplementary Note). The estimates scatter as expected around their weighted average (red dashed line with shaded region indicating theoretical s.e.m.). The latter value agrees with the true value of the parameter (black dashed line) within 2 per cent. The uncorrected values (blue circles) and their weighted average (blue dashed line with shaded region indicating theoretical s.e.m.) are shown for comparison. (**b**) Same as **a** for the product of the gain and the offset. (**c**) Same as **a** for the offset (Methods, Supplementary Fig. 2c). The weighted average agrees with the true value within 1 per cent. (**d-f**) Same as **a-c** for data simulated assuming a 2D-Gaussian PSF with parameters matching those of the PSF for the freely rotating fluorophore (Methods, Supplementary Fig. 1) and pixel output-signals distributed according to EMCCD camera statistics. The estimated values agree with the true values. (**g-i**) Same as **d-f** but assuming normally distributed output-signals (Supplementary Fig. 10). The estimated values agree with the true values. (**j-l**) Same as **g-i** but here the known true values of the expected pixel output-signals and calibration parameters were used in the correction for bias. There is no discernible difference between **j-l** and **g-i**, demonstrating that we introduce negligible error by using the fitted values for the expected pixel output-signals and the calibration parameters. (**m-o**) Same as **j-l** but using the known expected values of pixel output-signals in the fit. This artificially eliminates the need for the localization analysis and thus eliminates the bias introduced by that (Supplementary Note). The uncorrected values (blue circles) and their weighted average (blue dashed line with shaded region indicating theoretical s.e.m.) thus agree with the true value of the parameter (black dashed line).

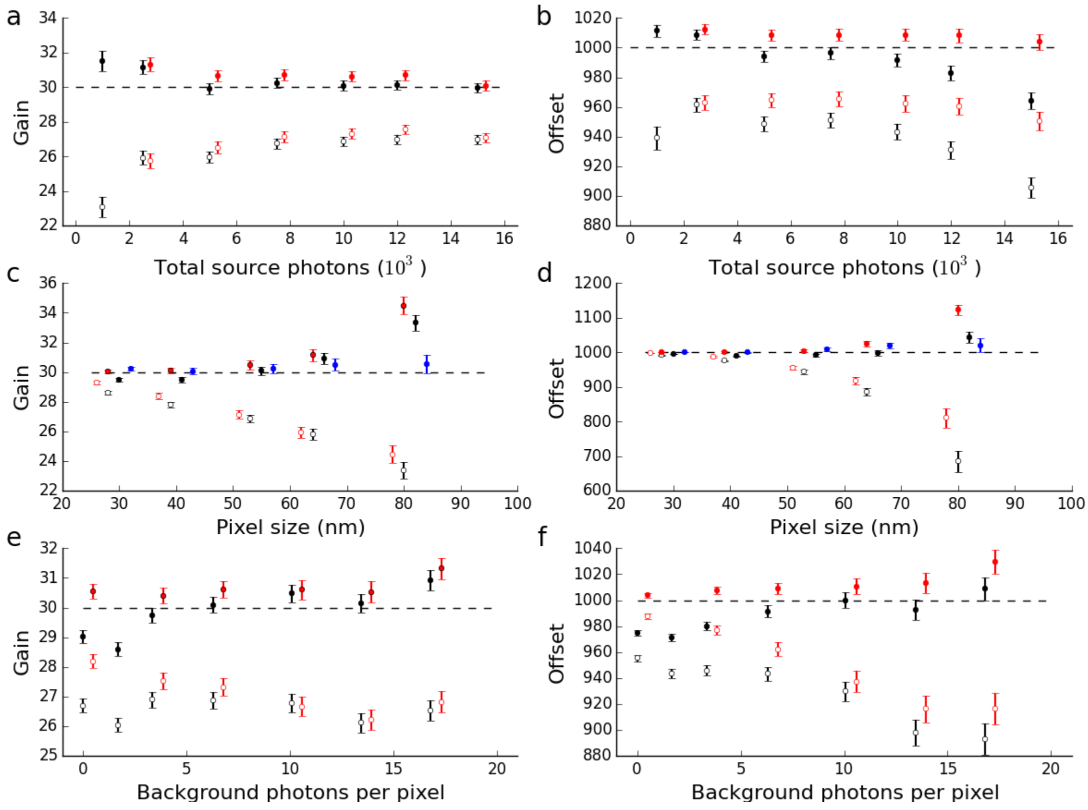

Supplementary Figure 5

Effect of source intensity, pixel size, and background level. We simulated images of isolated probes as described above (Methods, Supplementary Figs. 2 and 3). Specifically, we generated 100 images from each of ten probes and applied calibration-on-the-spot to each image to estimate the gain and offset. Their true values are indicated (dashed lines). (**a**) For various numbers of total expected source photons, the averaged gains with theoretical error bars are shown for, respectively, the true PSF for a freely rotating fluorophore (filled black circles) and a 2D-Gaussian PSF (filled red circles). In both cases, we simulated pixel output-signals using the full output-signal distribution (Methods, Eq. (1) in Supplementary Note) and corrected the estimates for bias (Methods, Supplementary Note). Uncorrected estimates are shown for comparison (open circles). Estimates agree with the known true value within a few per cent for all total source photon numbers. The pixel size was 55 nm and the background density was 0.002 photons/nm^2. (**b**) Same as **a** for the averaged offsets. For large total source photon numbers, the discrepancy between the PSF for a freely rotating fluorophore and the assumed 2D-Gaussian PSF results in a small bias. Note that this bias is absent for photons indeed distributed according to a 2D-Gaussian PSF. (**c**) For various pixel sizes, the averaged gains with theoretical error bars are shown for, respectively, the true PSF for a freely rotating fluorophore (filled black circles) and a 2D-Gaussian PSF (filled red circles). In the latter case, we used

normally distributed output-signals, while we corrected for bias in both cases. Uncorrected estimates are shown for comparison (open circles). Compare the first case to averaged gains (filled blue circles) obtained using the known true pixel output expected values in calibration-on-the-spot, such that no bias-correction is needed. This demonstrates that bias-correction works perfectly when pixels are small, as expected from theory (Supplementary Note). Here, its performance results in estimates that agrees with the known truth for pixel sizes smaller than ~70 nm. The total expected source photon number was 10,000 and the background density was 0.002 photons/nm^2. (**d**) Same as **c** for the averaged offsets. (**e**) Same as **a** for various numbers of expected background photons per pixel (of size 55 nm). The total expected source photon number was 10,000. For low background levels, the discrepancy between the PSF for a freely rotating fluorophore and the assumed 2D-Gaussian PSF results in a small bias. Note that this bias is absent for photons indeed distributed according to a 2D-Gaussian PSF. Despite this bias, the estimates agree with the known truth within a few per cent for all background levels. (**f**) Same as **e** for the averaged offsets.

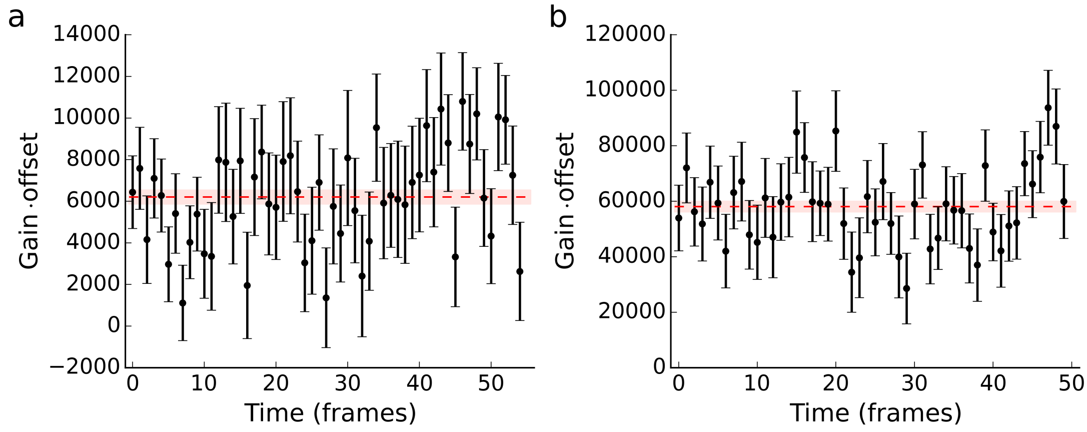

Supplementary Figure 6

Estimation of the product of gain and offset in calibration-on-the-spot. (**a**) For each frame in a time-lapse movie of a rhodamine fluorophore labelling a single myosin V stepping along actin as described in Fig. 2a, we simultaneously estimated the parameters of the EMCCD camera parameterized as the gain (Fig. 2a) and the product of the gain and offset (black points). The values were corrected for bias (Methods, Supplementary Note). Error bars represent the s.d. as calculated from the theoretical covariance matrix. We calculated the time-averaged value of the parameter and found $62\cdot10^2 \pm 3\cdot10^2$ (mean ± theoretical s.e.m., red dashed line with shaded area). (**b**) Same as **a** for the time-lapse movie of a single Cy3 fluorophore immobilized on the coverslip surface as described in Fig. 2b. In this case, we found $58\cdot10^3 \pm 2\cdot10^3$.

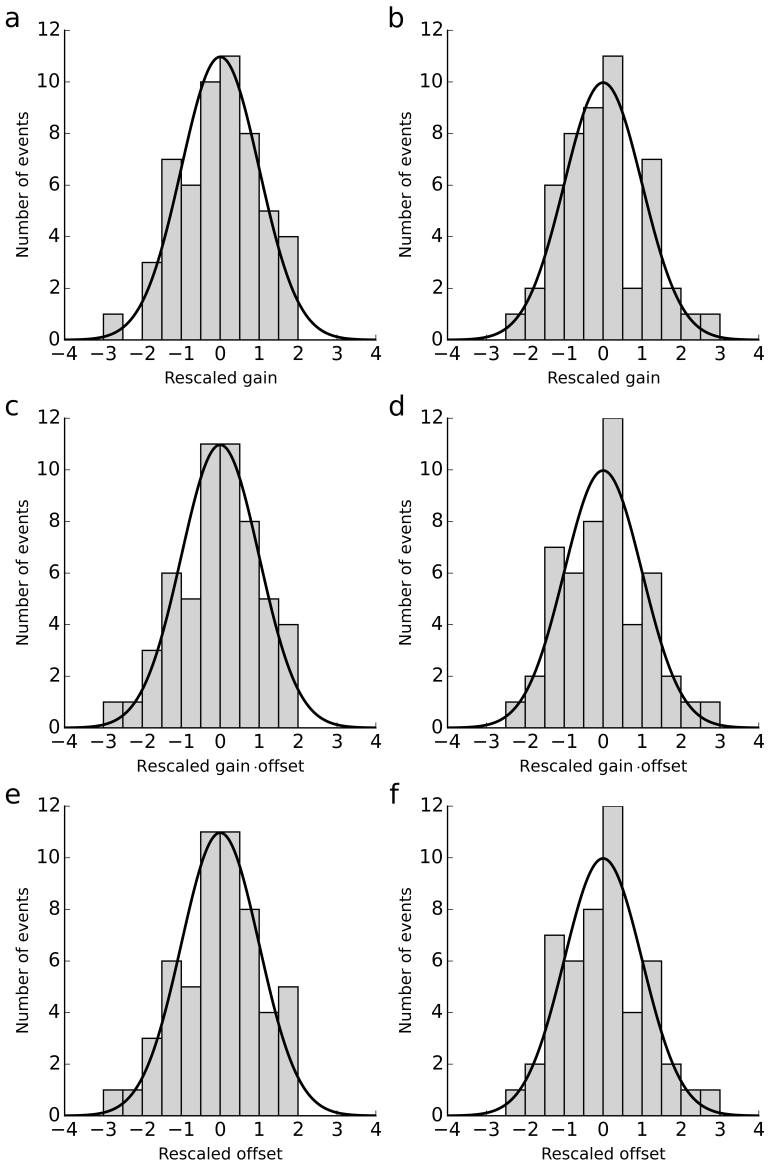

Supplementary Figure 7

Calibration-on-the-spot is optimally precise when applied to experimental data. (**a**) For the data in Fig. 2a from a single rhodamine fluorophore labelling a myosin V that was stepping along actin filaments, we subtracted the time-averaged gain from the frame-to-frame estimates of that quantity and rescaled each of those with their theoretical r.m.s. deviation. Their distribution follows a normal distribution with zero mean and unit variance (black curve), demonstrating that all variation in the estimates is accounted for by finite statistics and, thus, that calibration-on-the-spot is optimally precise. (**b**) Same as **a** for the data in Fig. 2b from a single Cy3 fluorophore immobilized on a coverslip surface. (**c**) Same as **a** for the product of the gain and the offset (Supplementary Fig. 6a). (**d**) Same as **b** for the product of the gain and the offset (Supplementary Fig. 6b). (**e**) Same as **a** for the offset (Fig. 2c). (**f**) Same as b for the offset (Fig. 2d).

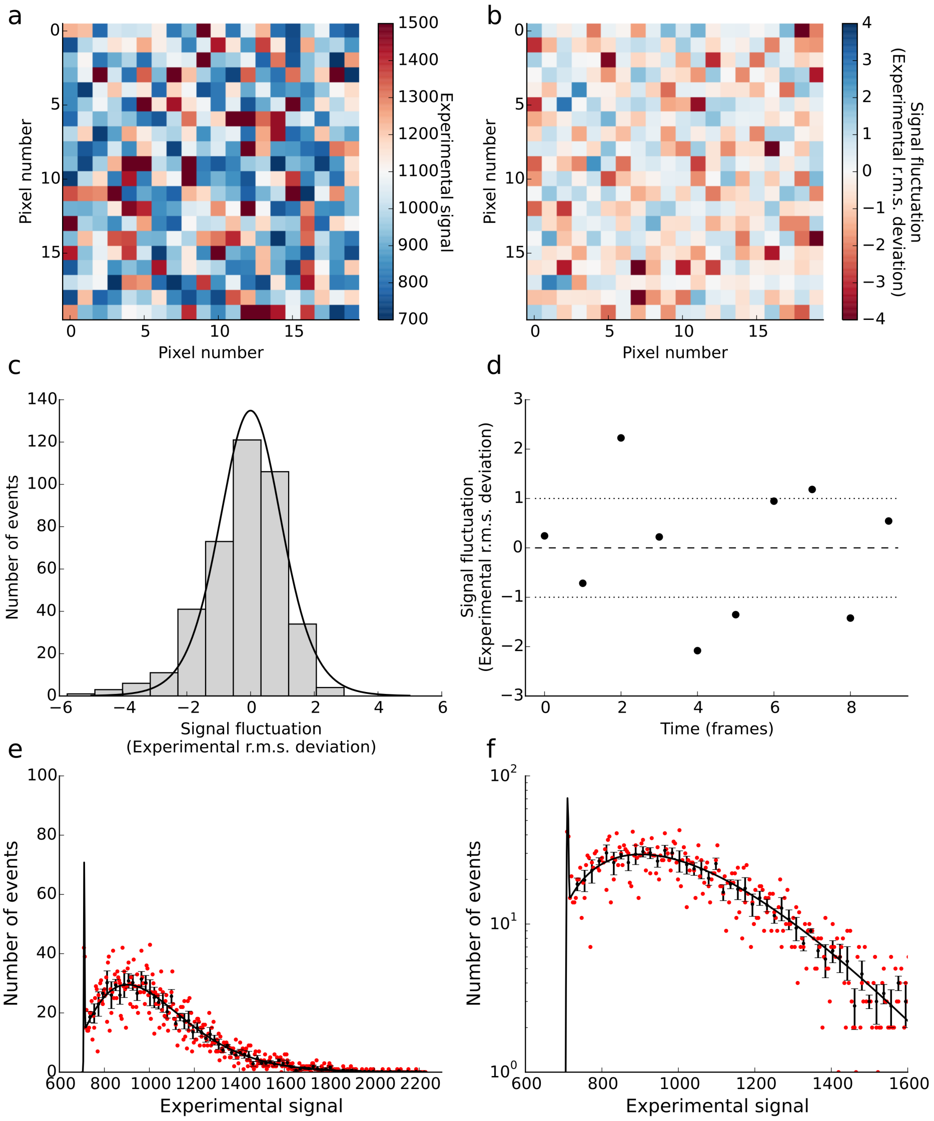

Supplementary Figure 8

Calibration using the full EMCCD output distribution. (**a**) Pixel output-signals in a region consisting of 20 x 20 pixels from a region in the field of view imaging background only selected from a time-lapse movie of immobilized Cy3 fluorophores (Methods, Fig. 2). (**b**) Fluctuations of time-averaged output-signals using ten consecutive frames from the time-lapse movie rescaled by the experimental r.m.s. deviation found from the pixel-wise sample variance. The fluctuations show no discernable spatial correlations, which indicates that all pixels experience the same expected number of input photons hence follow the same statistics. (**c**) Histogram of the rescaled fluctuations from **b**. Their distribution approximately follows Student's *t*-distribution with nine degrees of freedom (solid black curve). Note that the excess skewness, due to the discrepancy between the signal-output distribution of the EMCCD camera and the normal distribution (assumed in Student's *t*-distribution), does not affect the calibration below. (**d**) For each of the ten frames, we calculated

the fluctuations of region-averaged output-signals using all pixels in the region in **a** rescaled by the experimental r.m.s. deviation found from the frame-wise sample variance (black points). The points scatter approximately normal around zero (dashed line) with unit variance (dotted lines indicate ± s.d.) and show no discernable temporal correlations. (**e**) As all pixel output-signals in space and time follow the same statistics, demonstrated in **b-d**, we combined all measured output-signals. Their distribution (red points) follows the EMCCD output-signal distribution (black curve, Eq. 1 in Supplementary Note) with parameters obtained using maximum likelihood estimation (Methods). Specifically, we found a gain of 82, an offset of 710, an expected number of input photons of 4, and a readout-noise distribution width of 2. These numbers agree with those obtained using calibration-on-the-spot (Fig. 2f,h). Notice that the signature of the readout-noise is noticeable as a sharp peak around the value of the offset at this dim illumination; compare to Supplementary Fig. 10. We also calculated the average number of events from five consecutive bins in the histogram (black points, error bars are s.e.m.). Those points scatter as expected around the fitted theory. (**f**) Same as **e** but showing only a subset plotted with logarithmic second axis.

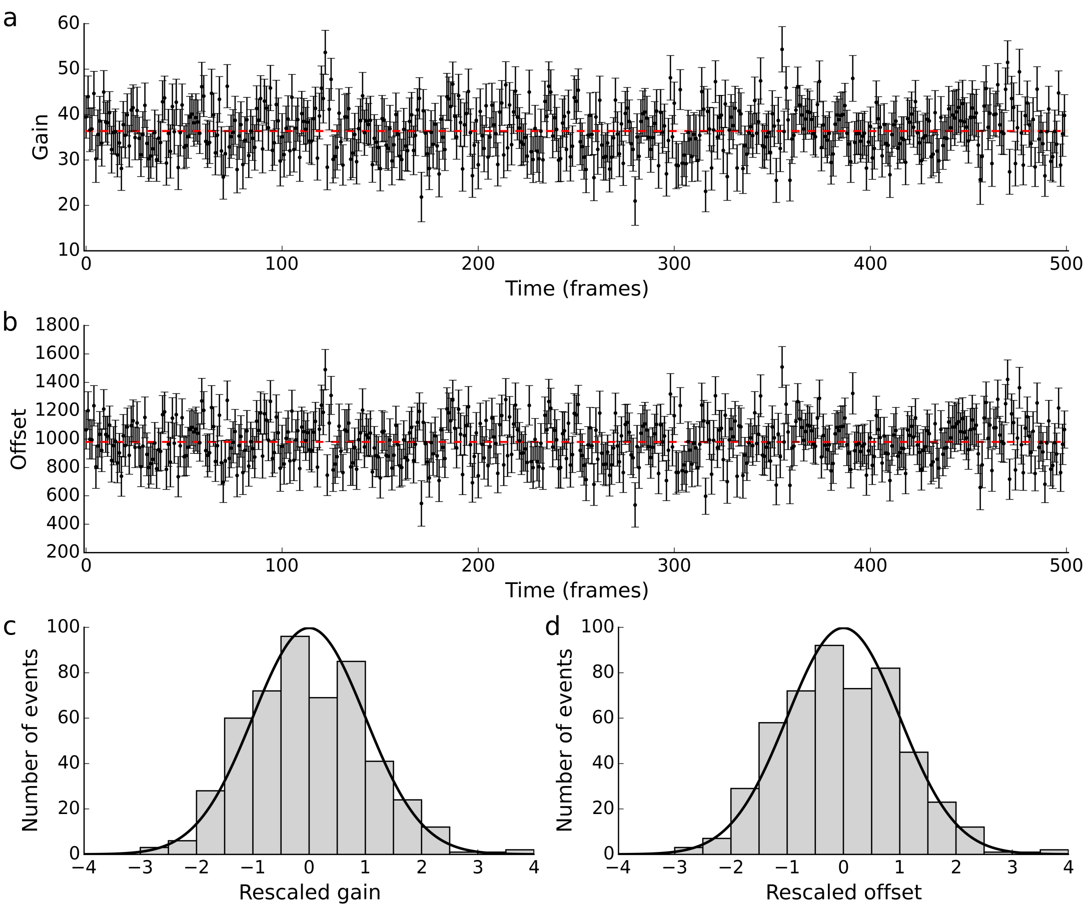

Supplementary Figure 9

Calibration-on-the-spot applied to an immobilized 40-nm fluorescent bead. (**a**) A single 40-nm fluorescent bead was imaged in a TIRF microscopy experiment as a time-lapse movie at 10 Hz. From each frame in the time-lapse movie, we used calibration-on-the-spot to estimate the gain (black points) of the EMCCD camera used in the experiment. Error bars represent the s.d. as calculated from the theoretical covariance matrix (Methods, Supplementary Note). The estimates were corrected for bias. We calculated the time-averaged gain and found 36.4 ± 0.2 (mean ± theoretical s.e.m., red dashed line with shaded area). (**b**) Same as **a** for the estimated offset of the EMCCD camera (black points). Here we found 980.5 ± 0.5 (mean ± theoretical s.e.m., red dashed line with shaded area). (**c**) For the data in **a**, we subtracted the time-averaged gain from the frame-to-frame estimates of that quantity and rescaled each of those with their theoretical r.m.s. deviation. Their distribution follows a normal distribution with zero mean and unit variance (black curve), demonstrating that all variation in the estimates is accounted for by finite statistics and, thus, that calibration-on-the-spot is optimally precise. (**d**) Same as **c** for the data from **b**.

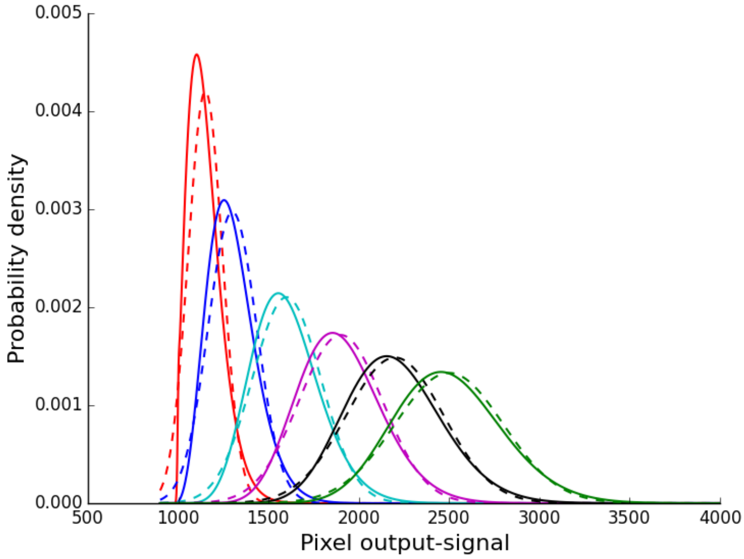

Supplementary Figure 10

The normal distribution approximates the EMCCD output-signal distribution. For expected input photon numbers of, respectively, 5 (red), 10 (blue), 20 (cyan), 30 (magenta), 40 (black), and 50 (green), the full evaluation of the EMCCD camera pixel output-signal distribution (Eq. (1) in Supplementary Note) is shown (full lines). For all evaluations, we used typical parameters for EMCCD cameras, specifically a gain of 30, a constant signal offset of 1,000, and a width of the readout-noise distribution of 5. Approximations of the full output-signal distribution by normal distributions with identical means and variances (Eqs. (2) and (3) in Supplementary Note) (dashed lines) become increasingly accurate with increasing expected input photon number. The discrepancies between the full evaluation and the approximation did not significantly compromise parameter estimation with calibration-on-the-spot (Fig. 2 and Supplementary Figs. 2-5).

SUPPLEMENTARY NOTE

1 Photon and EMCCD camera statistics

1.1 Photon input from an isolated, fluorescent probe

An isolated, fluorescent probe emits photons that are independent and identically distributed. When the probe is imaged in a localization microscopy experiment (Fig. 1a), the photons building the image arrive independently at random positions in the image plane with a probability density in the plane that equals the classical point spread function (PSF) of the experiment (Supplementary Fig. 1). The number n of photons detected by a given pixel in a single image of such a fluorophore (Fig. 1a and Supplementary Fig. 2a), is consequently a Poisson distributed random variable. We denote its expected value by ν: $\nu = \langle n \rangle$.

1.2 Pixel output-signals from an EMCCD camera

For each photon detected in a given pixel, the EMCCD camera's on-chip electron multiplication process outputs a random number S of electrons, where S has an exponential probability distribution $\exp(-S/G)/G$, with G the gain of the camera[1–4]. We assume that the gain is so large that we can treat that S as a *real* number. Consequently, n detected photons will output a random number of electrons that is drawn from the convolution of n identical exponential probability distributions $\exp(-S/G)/G$. This convolution results in the Erlang distribution with shape parameter n and rate parameter $1/G$. It then follows that the probability density p of output-signal values S generated in a pixel by Poisson distributed input photons with expected value ν, is

$$p(S) = \exp(-\nu)\delta(S) + \sqrt{\frac{\nu}{GS}} \exp(-S/G - \nu) I_1(2\sqrt{\nu S/G}) \ , \qquad (1)$$

where I_1 is the modified Bessel function of the first kind and of order one[1,4]. To model the effects of readout-noise of the camera, this distribution is convoluted with a Gaussian readout-noise distribution of width σ_{noise}. The presence of readout-noise makes it possible for the output-signals to take negative values. To ensure positive output-signal values everywhere, the output-signal is offset by a factory-set positive constant S_{offset}. By offsetting the above probability distribution with that amount, we have the theory for the output-signal from the EMCCD camera (Supplementary

Figs. 8 and 10). Even at low input intensity levels, the read-out noise of the camera is negligible for our purposes (Supplementary Figs. 2-5).

The first few central moments of S to leading order in G read

$$\langle S \rangle = G\nu + S_{\text{offset}} \tag{2}$$

$$\langle (S - \langle S \rangle)^2 \rangle = 2G^2\nu \tag{3}$$

$$\langle (S - \langle S \rangle)^3 \rangle = 6G^3\nu \tag{4}$$

$$\langle (S - \langle S \rangle)^4 \rangle = 12G^4\nu^2 + 24G^4\nu \ . \tag{5}$$

where $\langle \cdot \rangle$ denotes the expected value. Note that Eqs. (2) and (3) agree with the main paper's Eqs. (1) and (2).

When we apply calibration-on-the-spot, we take advantage of the fact that the pixel output-signal distribution, Eq. (1), approaches a normal distribution with mean and variance given by Eqs. (2) and (3) as the expected input signal ν increases (Supplementary Fig. 10).

1.3 Calibration using the EMCCD output distribution

One may calibrate with the EMCCD output distribution, Eq. (1), if one has access to pixel output-signals recorded with the EMCCD at spatiotemporal constant, dim illumination in the form of either a dedicated, separate calibration measurement[1,4] or the rather unlikely case where a region of constant background in actual recordings of fluorescent probes may be used (Fig. 2f,h and Supplementary Fig. 8). The first case requires additional measurements, and the latter presumably is only possible in the cleanest single-molecule experiments recorded with TIRF microscopy, while both require meticulous statistical verification of the assumption of constant illumination (Supplementary Fig. 8).

Using maximum likelihood estimation (MLE), one may use Eq. (1) to obtain the gain G, the constant signal offset S_{offset}, the width of the Gaussian readout noise distribution σ_{noise}, and the expected value of the constant photon input ν (Supplementary Fig. 8).

2 Localization analysis

2.1 Theory for image formation

We have compared methods for particle localization in fluorescence microscopy elsewhere[1], so we point the reader there for details. Thus, the following only contains information necessary to implement the localization analysis used in calibration-on-the-spot.

We assume that an isolated, fluorescent probe is imaged as a spatial intensity distribution for which we have a theory. The probability density for observation of a source photon at position $\vec{r}$ in an image is given by the point spread function $p_{\text{PSF}}(\vec{r}|\theta_{\text{PSF}})$, where θ_{PSF} is a set of parameters that specifies the PSF and typically includes the position coordinates of the fluorescent probe. The probability P_i to measure a source photon in the i'th pixel of the camera is

$$P_i(\theta_{\text{PSF}}) = \int_{\vec{r} \in \text{ pixel\#i}} d\vec{r}\, p_{\text{PSF}}(\vec{r}|\theta_{\text{PSF}}) \ . \tag{6}$$

Our theory for the expected value $E_i = \langle S_i \rangle$ of the output signal S_i in the i'th pixel on the camera in a single image is

$$E_i(\theta) = GNP_i(\theta_{\text{PSF}}) + Gb^2 + S_{\text{offset}} \ , \tag{7}$$

where N is the total expected number of detected source photons from the probe in a single image, hence GN is the total expected output signal due to source photons; and b^2 is the expected number of background photons detected with any pixel in each image, hence Gb^2 is the total expected pixel output signal due to the background. Here θ is a set of parameters to be estimated from data, which includes all parameters θ_{PSF} necessary to specify the PSF along with the total photon number, the constant background, and the calibration parameters.

2.2 Localization with the Gaussian Mask Estimator

Often, a 2D-Gaussian-plus-a-constant-background PSF is a very good approximation to the theoretical PSF of a localization microscopy experiment[1] (Methods, Supplementary Fig. 1), such as those investigated in this paper. Note, though, that notable exceptions exist[1,5]. In the case of a 2D-Gaussian PSF,

$$p(\vec{r}|\theta_{\text{PSF}}) = \frac{1}{\sqrt{2\pi\sigma_{\text{PSF}}^2}} e^{-(\vec{r}-\vec{\mu})^2/2\sigma_{\text{PSF}}^2} \ , \tag{8}$$

such that $\theta_{\rm PSF} = \{\vec{\mu}, \sigma_{\rm PSF}\}$ is comprised of the probe's position coordinates $\vec{\mu}$ and width $\theta_{\rm PSF}$ of the 2D-Gaussian PSF. With this choice, the set of parameters $\theta = \{\vec{\mu}, \sigma_{\rm PSF}, S, S_0\}$ specifies the expected output-signal in any pixel given by Eq. (7), where we have introduced the definitions $S_0 = Gb^2 + S_{\rm offset}$ and $S = GN$. These definitions highlight that localization performed by matching experimental and expected values of the pixel output-signal cannot determine the calibration parameters independently but only as functions of the unknown total expected photon number and background. For this very reason, the statistical properties of the pixel output-signals are unknown, hence cannot be taken into account in localization as pixel-dependent statistical weights of pixel outputs. Unweighted least-squares ignores pixel weights and results in the so-called Gaussian Mask Estimator (GME)[1,6], as discussed in the main paper. This estimator determines θ from the experimental signals $\{S_i\}_i$, where i labels each pixel included in the image, by minimizing the unweighted sum-of-squares,

$$\chi^2 = \sum_i (S_i - E_i(\theta))^2 \ , \tag{9}$$

with respect to the parameter set θ. By calculating the derivatives with respect each component θ_a in θ, we find a set of stationarity conditions[1],

$$\sum_i (S_i - E_i) E_{i,a} = 0 \ , \tag{10}$$

where $E_{i,a}$ denotes the derivative of E_i with respect to the a'th component of θ. In practice, this non-linear estimation is done numerically, by direct minimization of Eq. (9). Doing this, we obtain an estimate $\hat{\theta}$ for θ. In turn, we evaluate Eq. (7) at $\hat{\theta}$ to estimate the expected output-signal $\hat{E}_i = E_i(\hat{\theta})$ in each pixel, which we need to perform calibration-on-the-spot.

2.3 Linearization of the stationarity conditions

We denote the true value of the parameters by θ^*, and we assume that sufficient statistics have been acquired in an image to ensure that the deviation of our estimate $\hat{\theta}$ from θ^* is small. Note, however, that the deviations of the measured pixel output-signals S_i from their true values $E_i^* = E_i(\theta^*)$ are not necessarily small. The deviations from the true values of, respectively, the parameters and the pixel output-signals, we denote by

$$\Delta\theta = \hat{\theta} - \theta^* \tag{11}$$

$$\Delta S_i = S_i - E_i^* \ . \tag{12}$$

With $\Delta\theta$ assumed small, the fitted values $\hat{E}_i = E_i(\hat{\theta})$ for the expected pixel output-signal and its derivative $\hat{E}_{i,a} = E_{i,a}(\hat{\theta})$ with respect to the a'th component of θ may be written approximately as Taylor expansions to first order in $\Delta\theta$ around their true values,

$$E_i(\theta^* + \Delta\theta) = E_i^* + \sum_b E_{i,b}^* \Delta\theta_b \tag{13}$$

$$E_{i,a}(\theta^* + \Delta\theta) = E_{i,a}^* + \sum_b E_{i,a,b}^* \Delta\theta_b \ . \tag{14}$$

Here, $E_{i,a}^*$ denotes the derivative of E_i with respect to the a'th component in θ and evaluated at θ^*. When these relations are inserted into the stationarity condition, Eq. (10), the latter may be solved for the deviation in the a'th component of θ. Thus,

$$\Delta\theta_a = \sum_b (I^{-1})_{a,b} \sum_i \Delta S_i E_{i,b}^* \ , \tag{15}$$

where we have introduced the "information" matrix for GME,

$$I_{a,b} \equiv \sum_i E_{i,a}^* E_{i,b}^* \ . \tag{16}$$

Inserting Eq. (15) into Eq. (13), the fitted expected value for the pixel output-signal in the j'th pixel may be written as a function of the deviations in the pixel output-signals as

$$\begin{aligned} E_j(\hat{\theta}) &= E_j^* + \sum_b E_{j,b}^* \Delta\theta_b \\ &= E_j^* + \sum_i \Delta S_i \sum_{a,b} E_{j,a}^* (I^{-1})_{a,b} E_{i,b}^* \\ &\equiv E_j^* + \sum_i \Delta S_i f_{ij} \ , \end{aligned} \tag{17}$$

where we have defined

$$f_{ij} \equiv \sum_{a,b} E_{j,a}^* (I^{-1})_{a,b} E_{i,b}^* \ , \tag{18}$$

a matrix that describes the linear relationship between deviations of pixel output-signals from their expected values and deviations of fitted expected pixel values from their true values.

Note that this function is used in the analytical correction for bias of the estimated calibration parameters, as discussed below. Thus, if one wishes to perform bias-correction, the elements of f_{ij} should be calculated and stored, after having applied the localization analysis to an image of a spot. To do that, and for lack of knowledge about the true value θ^* of θ, we evaluate all occurrences of $E_{i,a}$ in Eqs. (16) and (18) at our estimated parameters $\hat{\theta}$.

3 Calibration-on-the-spot

We assume that the photon count in all pixels considered is sufficiently large to justify a normal distribution of output signals (Supplementary Fig. 10). This is typically the case because of actual photonic background and because we do not consider pixels in the tail of the PSF, but only in a square containing its central peak and shoulders (Supplementary Fig. 1). Thus, our theory for the output signal from the i'th pixel is a Gaussian distribution,

$$p_i(S_i|\theta_{\text{cots}}) = \frac{1}{\sqrt{2\pi v_i}} e^{-(S_i - E_i)^2/2v_i} \ , \tag{19}$$

in which the variance explicitly reads

$$v_i(\theta_{\text{cots}}) = 2G(E_i - S_{\text{offset}}) = 2GE_i - 2GS_{\text{offset}} \ . \tag{20}$$

We have already obtained estimates $\hat{E}_i$ for the expected values E_i in Eqs. (19) and (20) from the localization analysis. These expected values contain no information about the calibration parameters, as discussed above. Thus, all dependence on the calibration parameters $\theta_{\text{cots}} = \{G, S_{\text{offset}}\}$ in the signal output distribution in Eq. (19) is contained in its variance in Eq. (20). However, it is preferable to perform the calibration by determining, instead, the set of parameters

$$\theta_{\text{cots}} = \{G, GS_{\text{offset}}\} \equiv \{G, S'_{\text{offset}}\} \ , \tag{21}$$

the components of which appear linearly in Eq. (20). This choice ensures desirable convergence properties of the estimates below. We will use MLE to determine θ_{cots}. The likelihood for the set of parameters θ_{cots}, given the measured signals $\{S_i\}_i$, is

$$\mathcal{L}(\theta_{\text{cots}}|\{S_i\}_i) = \prod_i P_i(S_i|\theta_{\text{cots}}) \ , \tag{22}$$

and its logarithm is

$$\ell = \log\left(\mathcal{L}(\theta_{\text{cots}}|\{S_i\}_i)\right) = \sum_i - \log(v_i) - \frac{(S_i - E_i)^2}{v_i} \ . \tag{23}$$

The maximum likelihood estimate $\hat{\theta}_{\text{cots}}$ is found by maximization of the log-likelihood with respect to each of the components in θ_{cots}. For each component a in θ_{cots}, we have the stationarity condition

$$0 = \frac{\partial \ell}{\partial \theta_{\text{cots},a}} = \sum_i - \frac{v_{i,a}}{v_i} + \frac{(S_i - E_i)^2}{v_i^2} v_{i,a} \ , \tag{24}$$

where $v_{i,a}$ denotes the derivative of v_i with respect to the a'th component in θ_{cots}. In practice, the maximum likelihood estimates are obtained by numerical maximization of Eq. (23). Also note that, for lack of their true values, we will use the fitted expected pixel output-signals $\hat{E}_i$ in Eqs. (23) and (24) in place of E_i, when we perform this maximum likelihood estimation. This introduces a bias in the estimated parameters, as discussed in the main paper and below.

3.1 Linearization of the stationarity conditions

In the following, we will let the "cots" subscript on θ be implied, such that $\theta = \theta_{\text{cots}}$. Its true value is denoted θ^*. The deviation in each parameter estimate and the deviations in signals, respectively, from their true values may be written as

$$\Delta\theta_a = \hat{\theta}_a - \theta^*_a \tag{25}$$

$$\Delta S_i = S_i - E^*_i \ . \tag{26}$$

We will assume that the statistics of a recorded image is sufficiently good to ensure that the deviation $\Delta\theta_a$ of a the a'the calibration parameter estimate $\hat{\theta}_a$ from its true value θ^*_a is small. As above, the deviations of signal values from their true values are not necessarily small.

Our estimates for, respectively, the pixel specific variance $\hat{v}_i = v_i(\hat{\theta})$ and its derivative $\hat{v}_{i,a} = v_{i,a}(\hat{\theta})$ with respect to the a'th component in θ may be Taylor expanded to first order in $\Delta\theta$ around their true values $v^*_i = v_i(\theta^*)$ and $v^*_i = v_i(\theta^*)$,

$$v_i(\theta^* + \Delta\theta) = v^*_i + \sum_b v^*_{i,b}\Delta\theta_b \tag{27}$$

$$v_{i,a}(\theta^* + \Delta\theta) = v^*_{i,a} + \sum_b v^*_{i,a,b}\Delta\theta_b \ , \tag{28}$$

where $v^*_{i,a,b}$ is the derivative of $v_{i,a}$ with respect to the b'th component in θ and evaluated at θ^*. Now, inserting Eqs. (27) and (28) into the stationarity conditions, Eq. (24), yields

$$0 = \sum_i \left[\frac{1}{v^*_i + \sum_b v^*_{i,b}\Delta\theta_b} - \frac{(\Delta S_i - \sum_j f_{ij}\Delta S_j)^2}{(v^*_i + \sum_b v^*_{i,b}\Delta\theta_b)^2} \right] \left(v^*_{i,a} + \sum_b v^*_{i,a,b}\Delta\theta_b \right) \ . \tag{29}$$

Linearizing the terms in the square brackets with respect to $\Delta\theta$ gives us

$$0 = \sum_i \left[\frac{1}{v^*_i} \left(1 - \frac{1}{v^*_i} \sum_b v^*_{i,b}\Delta\theta_b \right) \right.$$
$$\left. - \frac{(\Delta S_i - \sum_j f_{ij}\Delta S_j)^2}{v^{*2}_i} \left(1 - \frac{2}{v^*_i} \sum_b v^*_{i,b}\Delta\theta_b \right) \right] \left(v^*_{i,a} + \sum_b v^*_{i,a,b}\Delta\theta_b \right) \ . \tag{30}$$

Keeping terms to first order in the parameter fluctuations, reduces the above to,

$$0 = \sum_i \left[\frac{v^*_{i,a}}{v^*_i} - \frac{v^*_{i,a}}{v^{*2}_i}(\Delta S_i - \sum_j f_{ij}\Delta S_j)^2 + \frac{1}{v^*_i}\sum_b v^*_{i,a,b}\Delta\theta_b - \frac{v^*_{i,a}}{v^{*2}_i}\sum_b v^*_{i,b}\Delta\theta_b \right.$$
$$\left. + \frac{2v^*_{i,a}}{v^{*3}_i}(\Delta S_i - \sum_j f_{ij}\Delta S_j)^2 \sum_b v^*_{i,b}\Delta\theta_b - \frac{1}{v^{*2}_i}(\Delta S_i - \sum_j f_{ij}\Delta S_j)^2 \sum_b v^*_{i,a,b}\Delta\theta_b \right] \ . \quad (31)$$

In the present case, with the parameterization introduced in Eq. (21), we have

$$v^*_{i,G} = 2E^*_i \quad (32)$$

$$v^*_{i,S'_{\text{offset}}} = -2 \ , \quad (33)$$

and thus $v^*_{i,a,b} = 0$, for $a, b \in \theta$, so the third and last terms of Eq. (31) vanish. Reorganizing the remaining terms, we find

$$\sum_b \sum_i \left[\frac{v^*_{i,a} v^*_{i,b}}{v^{*2}_i} - 2\frac{v^*_{i,a} v^*_{i,b}}{v^{*3}_i}(\Delta S_i - \sum_j f_{ij}\Delta S_j)^2 \right] \Delta\theta_b = \sum_i \left[\frac{v^*_{i,a}}{v^*_i} - \frac{v^*_{i,a}}{v^{*2}_i}(\Delta S_i - \sum_j f_{ij}\Delta S_j)^2 \right] \ . \quad (34)$$

Note that the deviations in pixel output-signals have zero expected value and are uncorrelated between different pixels, so

$$\langle \Delta S_i \rangle = 0 \quad (35)$$

$$\langle \Delta S_i \Delta S_j \rangle = v^*_i \delta_{ij} \ . \quad (36)$$

On the left-hand-side of Eq. (34), $\Delta\theta_b$ is a small quantity, so we approximate its coefficient with its value to leading order. Specifically, we replace linear and quadratic expressions in ΔS_i with their expected values to obtain

$$\sum_b \sum_i \left[\frac{v^*_{i,a} v^*_{i,b}}{v^{*2}_i} \left(-1 + 4f_{ii} - 2\frac{1}{v^*_i}\sum_j f^2_{ij} v^*_j \right) \right] \Delta\theta_b = \sum_i \left[\frac{v^*_{i,a}}{v^*_i} - \frac{v^*_{i,a}}{v^{*2}_i}(\Delta S_i - \sum_j f_{ij}\Delta S_j)^2 \right] \ . \quad (37)$$

Here we have used the relations

$$\left\langle \left(\sum_j f_{ij}\Delta S_j \right)^2 \right\rangle = \left\langle \sum_{j,k} f_{ij} f_{ik} \Delta S_j \Delta S_k \right\rangle = \sum_j f^2_{ij} v^*_j \ , \quad (38)$$

$$\left\langle \Delta S_i \sum_j f_{ij}\Delta S_j \right\rangle = \left\langle \sum_j f_{ij}\Delta S_i \Delta S_j \right\rangle = f_{ii} v^*_i \ . \quad (39)$$

By defining the matrix

$$I_{a,b} \equiv \sum_i \frac{v^*_{i,a} v^*_{i,b}}{v^{*2}_i} \left(1 - 4 f_{ii} + 2 \frac{1}{v^*_i} \sum_j f^2_{ij} v^*_j \right) \quad , \tag{40}$$

we now may write Eq. (37) as

$$\sum_b I_{a,b}\, \Delta\theta_b = \sum_i \left[\frac{v^*_{i,a}}{v^{*2}_i} (\Delta S_i - \sum_j f_{ij} \Delta S_j)^2 - \frac{v^*_{i,a}}{v^*_i} \right] \quad . \tag{41}$$

Thus, a deviation in an estimated calibration parameter can be explained in terms of deviations in pixel output-signal values as

$$\Delta\theta_a = \sum_b \left(I^{-1}\right)_{a,b} \sum_i \left[\frac{v^*_{i,b}}{v^{*2}_i} (\Delta S_i - \sum_j f_{ij} \Delta S_j)^2 - \frac{v^*_{i,b}}{v^*_i} \right] \quad . \tag{42}$$

3.2 Accuracy of estimators for calibration parameters

Accuracy quantifies agreement between an estimator's mean value and the true value it attempts to estimate. An accurate, or unbiased, estimator is thus characterized by $\langle \Delta\theta_a \rangle = 0$. By calculating the expected value of Eq. (42), we may directly quantify the bias of the calibration procedure,

$$\begin{aligned} \langle \Delta\theta_a \rangle &= \sum_b \left(I^{-1}\right)_{a,b} \sum_i \left[\frac{v^*_{i,b}}{v^{*2}_i} \left(v^*_i + \sum_j f^2_{ij} v^*_j - 2 f_{ii} v^*_i \right) - \frac{v^*_{i,b}}{v^*_i} \right] \\ &= \sum_b \left(I^{-1}\right)_{a,b} \sum_i \left[\frac{v^*_{i,b}}{v^{*2}_i} \left(\sum_j f^2_{ij} v^*_j - 2 f_{ii} v^*_i \right) \right] \quad . \end{aligned} \tag{43}$$

Thus, in general, estimates for the calibration parameters obtained as described in Sec. (3) are biased. This bias arises, because we are using the fitted expected pixel output-signals $\hat{E}_i$ obtained from the localization analysis in place of their true values E^*_i. Some of the variation in the pixel output-signals is thus absorbed by the parameters resulting from the localization analysis. Thus, when calibration is performed by analyzing the variation around $\hat{E}_i$ instead of E^*_i, one, e.g., underestimates the gain of the camera (Supplementary Figs. 4 and 5).

Had we, instead, known the true expected pixel output-signal values by other means than the localization analysis, we would have $f_{ij} = 0$ for all i, j by virtue of Eq. (17). In this special case, the estimators for the calibration parameters would be unbiased, because Eq. (43) shows that $\langle \Delta\theta_a \rangle = 0$ for all (both) a (Supplementary Figs. 4 and 5).

In practice, one does not know the true expected output-signals *a priori* and thus has to rely on the localization analysis to provide them, and the estimator will be biased in general. However,

with the analytical result of Eq. (43), one may correct estimated parameters for this bias (Fig. 2 and Supplementary Figs. 2—5 and 9). To this end, one uses the values of f_{ij} calculated and stored after the localization analysis and proceeds by evaluating all remaining terms in Eq. (43) at our estimates for the appropriate parameters instead of at their true values, for lack of this knowledge.

3.3 Precision of estimators for calibration parameters

While the *accuracy* of our estimators for calibration parameters is affected by our use of the fitted expected output-signals in place of their true values, the *precision* of our estimators is not (Fig. 2 and Supplementary Fig. 3). Neglecting this effect, we assume that $\hat{E}_i = E_i^*$ for all pixels i in an image of a spot, i.e. $f_{ij} = 0$ for all pixels i and j. In this approximation, a deviation in a parameter estimate, Eq. (42), becomes

$$\Delta\theta_a = \sum_b \left(I^{-1}\right)_{a,b} \sum_i \left[\frac{v_{i,b}^*}{v_i^{*2}} (\Delta S_i)^2 - \frac{v_{i,b}^*}{v_i^*} \right] \ , \tag{44}$$

with

$$I_{a,b} \equiv \sum_i \frac{v_{i,a}^* v_{i,b}^*}{v_i^{*2}} \ . \tag{45}$$

The variance of the estimator $\hat{\theta}_a$ may be written

$$\mathrm{var}\left(\hat{\theta}_a\right) = \left\langle (\hat{\theta}_a - \langle\hat{\theta}_a\rangle)^2 \right\rangle = \left\langle \Delta\theta_a^2 \right\rangle - (\langle\hat{\theta}_a\rangle - \theta_a^*)^2 \ . \tag{46}$$

For an unbiased estimator, $\langle\hat{\theta}_a\rangle = \theta_a^*$, and hence the last term in Eq. (46) vanishes. In the present case, there is in general a bias, so $\langle\hat{\theta}_a\rangle \neq \theta_a^*$, as discussed above. In an image with small pixels, the contribution from the bias is so small compared to the first term in Eq. (46) that one may safely ignore its contribution to the variance (Fig. 2 and Supplementary Fig. 3). In images with larger pixels, however, one cannot neglect the last term. It is estimated using the result for the bias in Eq. (43).

For any pixel size, calculation of the variance requires calculation of

$$\begin{aligned} (\Delta\theta_a)^2 = \left(I^{-1}\right)_{a,a}^2 \left[\sum_i \frac{v_{i,a}^*}{v_i^{*2}} (\Delta S_i)^2 - \frac{v_{i,a}^*}{v_i^*} \right]^2 + \left(I^{-1}\right)_{a,b}^2 \left[\sum_i \frac{v_{i,b}^*}{v_i^{*2}} (\Delta S_i)^2 - \frac{v_{i,b}^*}{v_i^*} \right]^2 \\ +2 \left(I^{-1}\right)_{a,a} \left(I^{-1}\right)_{a,b} \left[\sum_i \frac{v_{i,a}^*}{v_i^{*2}} (\Delta S_i)^2 - \frac{v_{i,a}^*}{v_i^*} \right] \left[\sum_i \frac{v_{i,b}^*}{v_i^{*2}} (\Delta S_i)^2 - \frac{v_{i,b}^*}{v_i^*} \right] \ , \end{aligned} \tag{47}$$

which has the expected value

$$\left\langle (\Delta\theta_a)^2 \right\rangle = \left(I^{-1}\right)^2_{a,a} \left[3\sum_i \frac{v^{*2}_{i,a}}{v^{*2}_i} - \left(\sum_i \frac{v^*_{i,a}}{v^*_i} \right)^2 + \sum_{i,j\neq i} \frac{v^*_{i,a} v^*_{j,a}}{v^*_i v^*_j} \right]$$
$$+ \left(I^{-1}\right)^2_{a,b} \left[3\sum_i \frac{v^{*2}_{i,b}}{v^{*2}_i} - \left(\sum_i \frac{v^*_{i,b}}{v^*_i} \right)^2 + \sum_{i,j\neq i} \frac{v^*_{i,b} v^*_{j,b}}{v^*_i v^*_j} \right]$$
$$+ 2\left(I^{-1}\right)_{a,a} \left(I^{-1}\right)_{a,b} \left[3\sum_i \frac{v^*_{i,a} v^*_{i,b}}{v^{*2}_i} - \left(\sum_i \frac{v^*_{i,a}}{v^*_i} \right) \left(\sum_i \frac{v^*_{i,b}}{v^*_i} \right) + \sum_{i,j\neq i} \frac{v^*_{i,a} v^*_{j,b}}{v^*_i v^*_j} \right] . \quad (48)$$

In practice, the variance associated with an estimate obtained with calibration-on-the-spot is found by evaluating each function in Eq. (48) at the estimated parameter values for lack of knowledge about their true values. A similar calculation leads to the covariance $\langle (\Delta\theta_a \Delta\theta_b) \rangle$ between estimates. While these expressions look complicated, their use is not: One simply plugs in values on the right-hand side and is done, since the left-hand side is the desired result. Computer code doing this is given in Supplementary Software. All the complications were in their derivation described here.

SUPPLEMENTARY REFERENCES

1. Mortensen, K. I., Churchman, L. S., Spudich, J. A. & Flyvbjerg, H. Optimized localization analysis for single-molecule tracking and super-resolution microscopy. *Nat. Methods* **7,** 377–381 (2010).

2. Hynecek, J. & Nishiwaki, T. Excess noise and other important characteristics of low light level imaging using charge multiplying CCDs. *IEEE Trans. Electron Devices* **50,** 239–245 (2003).

3. Robbins, M. S. & Hadwen, B. J. The noise performance of electron multiplying charge-coupled devices. *IEEE Trans. Electron Devices* **50,** 1227–1232 (2003).

4. Ulbrich, M. H. & Isacoff, E. Y. Subunit counting in membrane-bound proteins. *Nat. Methods* **4,** 319–321 (2007).

5. Mortensen, K. I., Sung, J., Flyvbjerg, H. & Spudich, J. A. Optimized measurements of separations and angles between intra-molecular fluorescent markers. *Nat. Commun.* 6:8621 (2015). doi:10.1038/ncomms9621

6. Thompson, R. E., Larson, D. R. & Webb, W. W. Precise nanometer localization analysis for individual fluorescent probes. *Biophys. J.* **82,** 2775–2783 (2002).
