# Supplementary material for: “Calibration-on-the-spot”: How to calibrate an EMCCD camera from its images
Source: Sci Rep. 2016 Jul 6;6:28680. doi: 10.1038/srep28680 (PMC4933971; doi:10.1038/srep28680)
Supplement: SupplementarySoftware_readme [file srep28680-s2.pdf]

SUPPLEMENTARY SOFTWARE: README

for

"Calibration-on-the-spot": How to calibrate an EMCCD camera from its images

Kim I. Mortensen[+] and Henrik Flyvbjerg[+]

[+] Department of Micro- and Nanotechnology, Technical University of Denmark, Kongens Lyngby, DK-2800, Denmark

Supplementary Software contains four files:

1. SupplementarySoftware_readme.pdf
2. SupplementarySoftware.py
3. SupplementarySoftware_test.py
4. SupplementarySoftware_testdata.tif

The enclosed Python (python.org) code was written and tested with the following versions of Python and required libraries:

Python (2.7.9)
matplotlib (1.4.3)
scipy (0.15.1)
numpy (1.9.2)
PIL (1.1.7)

SupplementarySoftware.py contains code necessary for performing the analysis of calibration-on-the-spot, i.e. maximum likelihood estimation of the calibration parameters of an EMCCD camera based on isolated diffraction-limited spots, including code for the localization of such spots using unweighted least-squares, and the calculation of the theoretical bias and covariance matrix for the calibration estimates. In this module, the class *COTS* contains the functions that do this: Input data should be supplied as a tif-image (single image) or -stack (time-lapse movie) of EMCCD camera pixel output-signals. The data should contain the diffraction-limited image of at least one fluorescent probe. The function *Track* then uses the function

Estimate and the class *ChiSquare* to perform the unweighted least-squares estimation of the probe's position coordinates, the width of the point spread function, the background intensity per pixel in the image, and the total source intensity, for each frame in the time-lapse movie of the spot. The function *Signals* then calculates the fitted expected output-signal values. Finally, the function *Calibrate* determines the calibration parameters of the EMCCD camera using calibration-on-the-spot, and it calculates the theoretical bias and the covariance matrix of the estimates. *Calibrate* outputs the estimated calibration parameters averaged over all frames included in the analysis along with the theoretical error bars for these estimates.

SupplementarySoftware_test.py contains an example script detailing the use of *SupplementarySoftware.py*. It analyses *SupplementarySoftware_testdata.tif* that contains a time-lapse movie of five frames of simulated EMCCD pixel output-signals from a single fluorophore free to rotate during imaging. To submit a specific spot in an image for calibration-on-the-spot analysis, initial values for the spot's position and for parameters of its point spread function must be specified along with an initial guess for the EMCCD calibration parameters.

The code is licensed under a Creative Commons Attribution 4.0 International License. To view a copy of this license, visit http://creativecommons.org/licenses/by/4.0/
